# Predicting Patients' Satisfaction With Mental Health Drug Treatment Using Their Reviews: Unified Interchangeable Model Fusion Approach

**DOI:** 10.2196/49894

**Published:** 2023-12-05

**Authors:** Yi Wang, Yide Yu, Yue Liu, Yan Ma, Patrick Cheong-Iao Pang

**Affiliations:** 1 Faculty of Applied Sciences, Macao Polytechnic University Macao Macao; 2 School of Computer Science Beijing University of Posts and Telecommunications Beijing China

**Keywords:** artificial intelligence, AI, mental disorder, psychotherapy effectiveness, deep learning, machine learning, natural language processing, NLP, data imbalance, model fusion

## Abstract

**Background:**

After the COVID-19 pandemic, the conflict between limited mental health care resources and the rapidly growing number of patients has become more pronounced. It is necessary for psychologists to borrow artificial intelligence (AI)–based methods to analyze patients’ satisfaction with drug treatment for those undergoing mental illness treatment.

**Objective:**

Our goal was to construct highly accurate and transferable models for predicting the satisfaction of patients with mental illness with medication by analyzing their own experiences and comments related to medication intake.

**Methods:**

We extracted 41,851 reviews in 20 categories of disorders related to mental illnesses from a large public data set of 161,297 reviews in 16,950 illness categories. To discover a more optimal structure of the natural language processing models, we proposed the Unified Interchangeable Model Fusion to decompose the state-of-the-art Bidirectional Encoder Representations from Transformers (BERT), support vector machine, and random forest (RF) models into 2 modules, the encoder and the classifier, and then reconstruct fused “encoder+classifer” models to accurately evaluate patients’ satisfaction. The fused models were divided into 2 categories in terms of model structures, traditional machine learning–based models and neural network–based models. A new loss function was proposed for those neural network–based models to overcome overfitting and data imbalance. Finally, we fine-tuned the fused models and evaluated their performance comprehensively in terms of *F*_1_-score, accuracy, κ coefficient, and training time using 10-fold cross-validation.

**Results:**

Through extensive experiments, the transformer bidirectional encoder+RF model outperformed the state-of-the-art BERT, MentalBERT, and other fused models. It became the optimal model for predicting the patients’ satisfaction with drug treatment. It achieved an average graded *F*_1_-score of 0.872, an accuracy of 0.873, and a κ coefficient of 0.806. This model is suitable for high-standard users with sufficient computing resources. Alternatively, it turned out that the word-embedding encoder+RF model showed relatively good performance with an average graded *F*_1_-score of 0.801, an accuracy of 0.812, and a κ coefficient of 0.695 but with much less training time. It can be deployed in environments with limited computing resources.

**Conclusions:**

We analyzed the performance of support vector machine, RF, BERT, MentalBERT, and all fused models and identified the optimal models for different clinical scenarios. The findings can serve as evidence to support that the natural language processing methods can effectively assist psychologists in evaluating the satisfaction of patients with drug treatment programs and provide precise and standardized solutions. The Unified Interchangeable Model Fusion provides a different perspective on building AI models in mental health and has the potential to fuse the strengths of different components of the models into a single model, which may contribute to the development of AI in mental health.

## Introduction

### Background

According to the World Health Organization, the number of people who have had anxiety and depressive illnesses has greatly increased since 2020 owing to the COVID-19 pandemic and modern high-paced lifestyles. The early projections indicate a 26% and 28% increase in anxiety and severe depressive disorders, respectively, in 2020 [1]. Moreover, a study has shown that people with mental illnesses may be socially ostracized, stigmatized, or discriminated against [2]. Mental health has become a crucial issue for global development as it affects millions of people worldwide and has a significant social and economic impact. Therefore, there is an urgent need to address the challenges and gaps in mental health care and to promote efficient treatment. The treatment of mental illness implies a long-term process for some patients with mental illnesses. During this process, a psychologist must frequently judge the effectiveness or satisfaction of psychotherapy and drug therapy at a certain stage based on the patient’s feedback and their professional skills. The judgment can help psychologists to monitor the changes in the patient’s condition for adjusting the treatment plan accordingly. Moreover, it is necessary to identify and repair any ruptures or conflicts that may arise in the therapeutic relationship. Psychologists can then empower the patient to take an active role in their own recovery and enhance their motivation and satisfaction with the therapy. For instance, psychologists often use patient feedback to measure the effectiveness of the treatment approach [3-6]. However, such a manual analysis conducted by psychologists is sometimes inefficient and inconsistent across different psychologists. Moreover, a large amount of jumbled information provided by patients with mental illness may lead to neglected critical information, possibly negatively affecting the reliability of the analysis process. The analysis process requires a large amount of human resources. Coupled with the fact that medical resources are insufficient in many locations, there may be many patients with mental illnesses who do not receive effective treatment and whose feedback cannot be analyzed in a timely manner. Deep learning (DL) and machine learning (ML) in natural language processing (NLP) can handle large amounts of electronic health records to extract information quickly [7]. Hence, they have the potential to improve the efficiency of mental health care decision-making, alleviate insufficient medical resources, and shed light on solutions to address these issues [7].

### Related Work

In NLP, there are massive methods for input presentations that generate information containing suitable context, dimensionality, and feature types to ensure the accurate prediction of a classification model [8]. Patient review analysis can seek the opinions or perspectives of patients with mental disorders by extracting specific emotional expressions from their comments [9]. This is regarded as an effective way to evaluate the state of patients. Simple NLP methods such as bag-of-words (BOW) [10] convert text into fixed-length vectors by counting the frequency of each word. Its variant, term frequency–inverse document frequency (TF-IDF) measure [11], is a weighting scheme that assigns a score to each word in a document based on its term frequency and inverse document frequency. TF-IDF enhances the term frequency BOW vectors by assigning more weight to relevant words and less weight to common words [12], and it can be effectively used in different applications. These methods offer significant advantages in terms of speed. However, they disregard the context, and their performance may be unsatisfactory for the tasks in which contextual information is significant for prediction [13,14]. Recently, transformer-based [15] models were introduced, and the state-of-the-art method in this series is Bidirectional Encoder Representations from Transformers (BERT) [16]. They can embed the contextual information of the text and have the potential to evaluate patients’ feedback.

In addition, sentiment classification NLP models can be divided into several main categories: symbolic artificial intelligence (AI) and rule-based systems, traditional ML based, and DL based. For traditional ML-based models, support vector machine (SVM) [17-19] and tree models such as random forest (RF) [20] and decision trees are representative. In contrast, popular DL-based models include but are not limited to recurrent neural network (RNN) [21], convolutional neural network (CNN) [22], and attention-based network [15,23]. These methods can address the challenges and gaps in the field of mental health care.

The recent development of thriving NLP technologies has been applied to many medical-related tasks. For instance, a medical opinion lexicon deals with the health care problem of patients [24], a health care analysis–based study on medicines and services [25], an analysis of electronic health records and chatbots for patient communication [26], and NLP methods for extracting information from radiology reports [27]. According to the study by Le Glaz et al [28], traditional ML methods and classical neural networks have been heavily used in mental health domain in previous studies and the informality of text data on mental health was exposed in previous studies. ML-based and DL-based methods have been proven effective and used extensively for mental disorder detection, especially for depression and suicide [29]. A long short-term memory (LSTM)–based RNN [30] and a hybrid CNN and LSTM model [31] were used to detect depression. Besides this, Shah-Mohammadi et al [32] measured treatment effectiveness by discharge records and discharge status. Clinical Language Annotation, Modeling and Processing was used to extract entities from the patients’ notes, and RF and logical regression were applied to predict treatment effectiveness. In addition, Zhang et al [33] used 8000 attention-deficit/hyperactivity disorder medication prescriptions to train 2 dense neural network models: one for identifying noninformative prescription texts and the other for predicting prescribed daily dosage and treatment duration. The metrics used to evaluate the models were accuracy, precision, recall, and *F*_1_-score [29]. However, they do not consider the large variance in model performance for multiclass classification owing to data imbalance. The DL or ML models trained on drug reviews in the health care field mainly deal with text on physiological aspects such as relief of pain and breathing difficulty, rather than focusing on patients’ psychological states. Moreover, the way in which patients with mental disorders express themselves might be very different from that of patients with physical illnesses. For example, for depression, the analysis should focus on whether a patient feels cheerful after taking the drug; however, for gastric colic, it is more about whether the pain is relieved. As such, we cannot use the same standard to measure the reviews of physiological treatment and psychological treatment.

### Significance of the Study

This paper pilots applying burgeoning AI technologies to analyze patients’ feedback to help psychologists to evaluate patients’ satisfaction with drug treatment to rapidly adapt the medication regimen. In this paper, we proposed a model fusion method called Unified Interchangeable Model Fusion (UIMF) to decompose SVM, RF, and BERT models and recompose 6 fused models. Then, we trained them on an open data set consisting of the reviews and scores of the drug treatment provided by patients with mental disorders. A novel loss function was proposed for all neural network–based models to alleviate overfitting and data imbalance problems. In addition, we adopted the κ coefficient [34-36], a metric that accounts for chance agreement in imbalanced data, along with other general metrics, to comprehensively evaluate the model performance. The results revealed that the fused transformer bidirectional encoder+RF model performs the best performance while consuming more computing resources and shows a high degree of reliability and indicates that the prediction results of the model are almost in perfect agreement with the random choice of patients’ subjective satisfaction. The word-embedding encoder+RF model requires the least computing resources and exhibits relatively good performance.

## Methods

### Data

#### Overview

We combined 2 open medical-related text data sets on Kaggle [37,38] to form the raw data set. It contained 16,950 categories of physical and mental illnesses. For each review, 4 attributes, including patient ID, subjective scores (1-10), condition, and drug name are provided. Table 1 provides several sample data.

**Figure 1:**
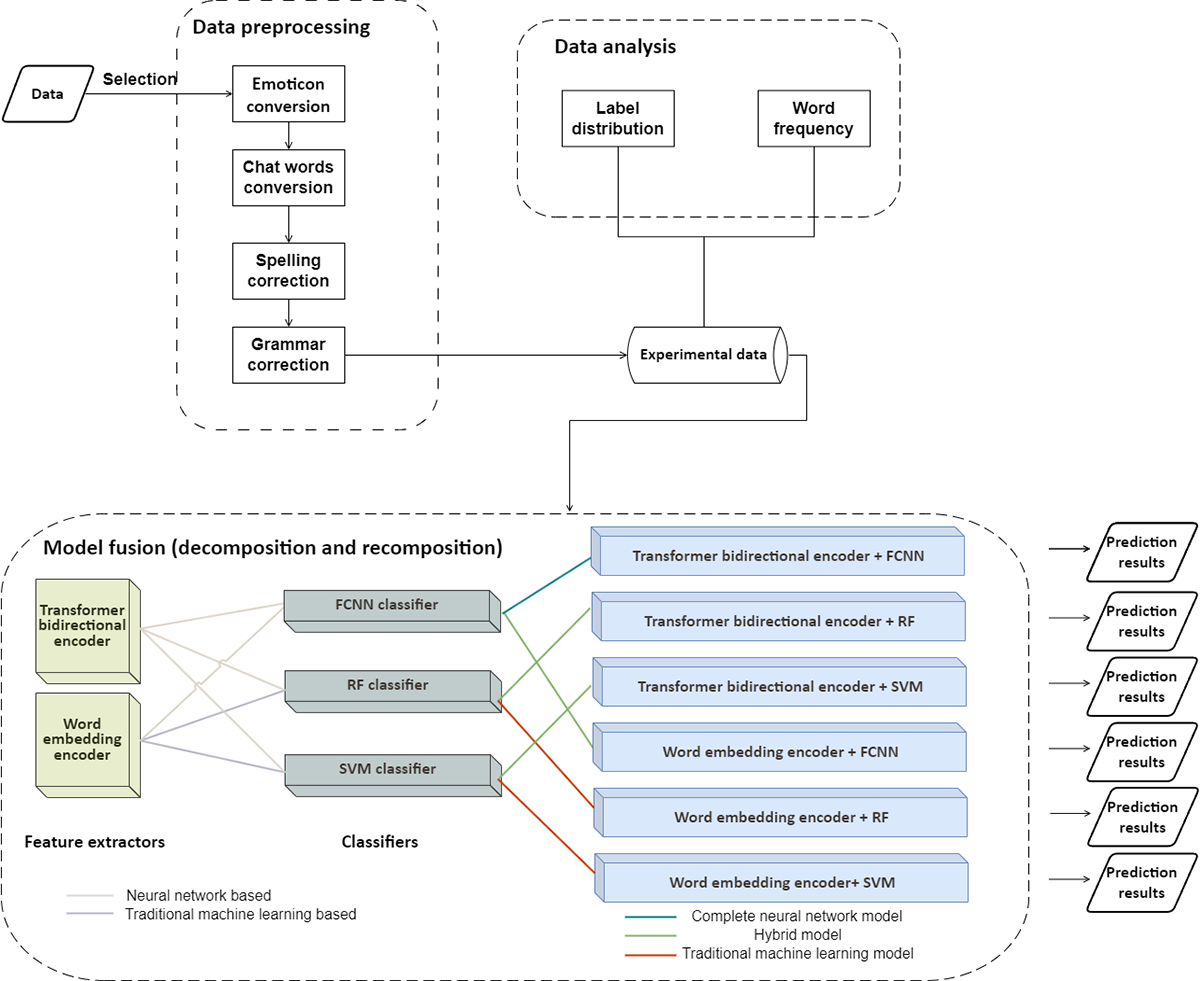
Overview of our workflow: data selection, data preprocessing, data analysis, and Unified Interchangeable Model Fusion. FCNN: fully connected neural network; RF: random forest; SVM: support vector machine.

Figure 1 illustrates the workflow of the operations consisting of data handling and model fusion. Data handling includes 3 main tasks: data selection, data preprocessing, and data analysis.

**Table 1:** Sample data of the 2 open medical-related text data sets on Kaggle.

Unique ID	Drug name	Condition	Review	Score
206,461	Valsartan	Left ventricular dysfunction	“It has no side effect, I take it in combination of Bystolic 5 Mg and Fish Oil”	9
92,703	Keppera	Epilepsy	“I Ve had nothing but problems with the Keppera: constant shaking in my arms & legs & pins & needles feeling in my arms & legs severe light headedness no appetite & etc.”	1
121,333	Venlafaxine	Depression	“My go started me on venlafaxine yesterday to help with depression and the change, a hour after taking them I was feeling very sick couldn’t stomach food or fluids, thought keep it up as she told me they did come with some side effects which would get better, took another one last night and was so ill I couldn’t stand, being sick sweating shaking thought I was going to pass out. Did get some sleep hoping to feel better this morning, took another one and felt so spaced-out dry mouth shaking, sick, so booked in to see go again to make sure I should be feeling like this, only to find out she had put me on the wrong dose should have been on 37.5mg was put on 150mg, now on right dose hope this will be better”	4

#### Data Selection

To create the target data set, we extracted 19 categories of mental disorders defined by the World Health Organization [39] and insomnia which often coincides with the diagnosis of mental disorders [40] from the Kaggle data sets. The target data set included bipolar disorder, depression, panic disorder, psychosis, schizophrenia, anxiety, major depressive disorder, obsessive compulsive disorder, generalized anxiety disorder, autism spectrum disorder, paranoid disorder, performance anxiety, schizoaffective disorder, agitated depression, social anxiety disorder, postpartum depression, dissociative identity disorder, persistent depressive disorder, intermittent explosive disorder, hyperekplexia, and insomnia. The target data set comprised 41,851 reviews.

#### Data Preprocessing

When writing the review, patients would inevitably use emojis and web-based chat buzzwords and make grammatical and spelling errors, which can result in the introduction of irrelevant symbols and noise that interfere with the important emotional information. In addition, some garbled codes or errors might be introduced owing to human or irresistible factors in data collection. Therefore, we applied a data preprocessing process that sequentially performed emoticon conversion, chat words conversion, spelling correction, and grammar correction. The process aimed to reduce the influence of these factors on the classification performance and to reduce the complexity and dimensionality of the original data. The details of the data preprocessing are listed in subsequent sections.

Emoticons conversion transforms emoticons into their textual meanings. Unlike normal punctuations with weaker emotional polarity, emoticons always represent strong emotions. To preserve the emotional information represented by the emoticons, a Python dictionary of emoticons EMOTICONS_EMO [41] was used to translate emoticons into textual meanings.

Chat words conversion transforms slang, informal, or nonstandard language into standard text. These words can convey various emotions and always have a clear sentiment polarity. However, some NLP corpus or lexicons may not include or only partially include them, leading to the missing emotional state of the patient. In addition, a consistent and clear style of writing is required in the patients’ sentiment analysis. We constructed a Python dictionary based on the slang data [42] from GitHub, which consists of popular slang and abbreviations to convert chat words.

The NLP analysis of the health care domain is sensitive to spelling and grammar mistakes, as these errors can significantly affect the quality and credibility of AI-based health care prediction methods [43]. Confusion and misunderstanding may occur between the patient and psychological therapist, which can result in potential errors or risks in diagnosis and treatment. In a worse-case scenario, training a model with data that contain a large number of spelling and grammar errors may introduce bias and noise in the results, which can affect critical health care decision-making. Therefore, we have applied spelling and grammar corrections to this study. For spelling correction, the algorithm by Peter [44] was used to adjust the filtering conditions for candidate words. Two editing distances [45] were used to restrict the difference between the misspelt word and candidate word. The Python API [46] for grammar correction was chosen after conducting a feasibility assessment.

**Figure 2:**
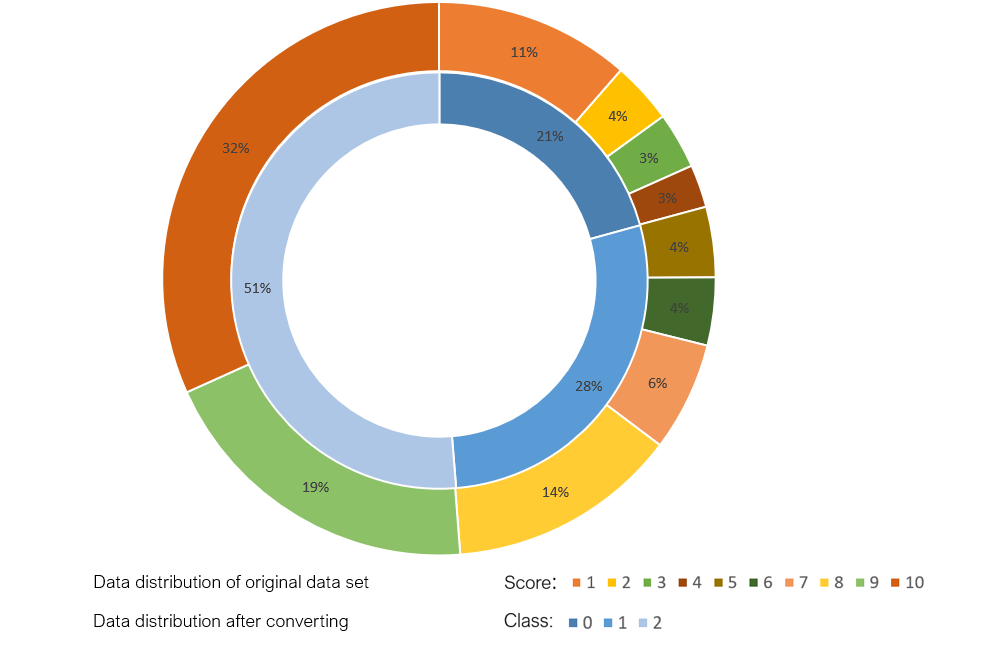
Data amount for 10 scores and for the 3 classes.

The label distribution analysis in Figure 2 shows the distribution of data across different scores. It can be seen that the data set with 10 scores encounters a more severe data imbalance problem. Data imbalance normally affects the variance in classification performance. It will lead the model to produce higher accuracy for scores with more data and vice versa. To alleviate this problem while considering the actual need for classification, we converted the 10 scores into 3 classes with labels 0, 1, and 2 for poor, fair, and good comments, respectively. As shown in Figure 2, the data distribution of the 3 classes became more balanced after the conversion. Table 2 shows the mapping between the 10 scores and 3 classes.

**Table 2:** The rules of label mapping and the meaning of each class label.

Class label	Score range	Proportion of data (%)	Meaning
0	1-4	20.82	A poor satisfaction of drug treatment and case needs urgent rediagnosis.
1	5-8	27.96	A fair satisfaction of drug treatment and cases needs to be monitored.
2	9-10	51.22	A good satisfaction of drug treatment and no further action needed.

For the target data set, we extracted 30% of the data for testing, 63% for training, and 7% for validation by stratified sampling and used a 10-fold validation strategy for training.

### Problem Formulation

#### Overview

For any patient, the review data were 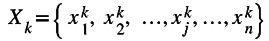, the score given by the patient for the treatment was 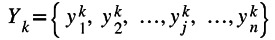, where 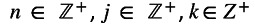, 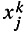 and 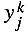 were the review record and the corresponding score provided by the patient during the *j^th^* treatment, respectively. Here we regarded each review and score pair as independent. The score was only affected by the corresponding review.

For each review 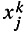 the loss function was defined as 
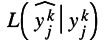
, 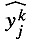 was the prediction result of a prediction function *f*, and 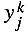 was the ground truth of 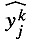. As the prediction function is decomposed into encoder function and classification function, it is denoted as follows:



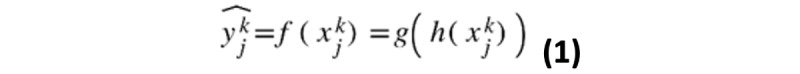



where 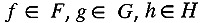, and *F* are the set of fused prediction functions of *G*; *H*, *G*={RF, SVM, fully CNN (FCNN)} is the set of classification functions; and *H*={transformer bidirectional encoder, word-embedding encoder} is the set of encoder functions.

The objective of this study was to find the optimal recomposed prediction function 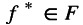 to minimize the empirical risk.



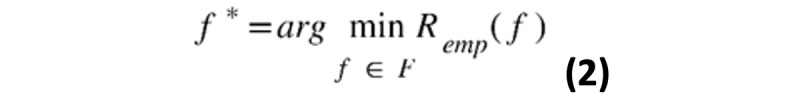



where *R_emp_(f)* is the empirical risk, which is a concept in statistical learning theory that measures how well a learning algorithm performs on a given data set [47]:



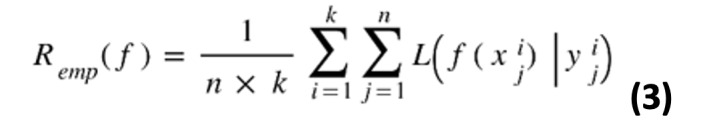



#### Preliminary Data Analysis

In Textbox 1, we show the top 30 most frequent words in class 2 (a good satisfaction), class 1 (a fair satisfaction), and class 0 (a poor satisfaction). It shows that the high-frequency words in the 3 classes are extremely similar. If using the BOW method that transforms a given text into a vector based on the word frequency, the vectors of those similar high-frequency words will have higher values in more dimensions and vice versa. In this situation, the vectors of high-frequency words become more critical influencing the classification than the low-frequency words. However, low-frequency words do not mean that they are always irrelevant or useless to the text; they may capture some specific or rare information. Hence, the term frequency BOW method could result in text vectors from every class being similar to each other for classifiers, and the distinctive information of text vectors from different classes is buried. Using TF-IDF helps distinguish the text from each class by reducing the vector similarity and highlighting the unique features. Therefore, we applied TF-IDF to the vectorizer to measure the importance of words in a text review and weighted the embedding of text based on word importance. The details of this are discussed in the following section.

**Top 30 most frequent words in 3 classes (the high-frequency words shared by all 3 classes are in italics).**

**Top 30 most frequent words in class 0**

- also
- anxiety
- day
- depression
- doctor
- drug
- effect
- feel
- felt
- first
- get
- help
- hour
- like
- made
- medication
- month
- night
- one
- side
- sleep
- started
- take
- taking
- time
- took
- week
- work
- would
- year

**Top 30 most frequent words in class 1**

- also
- anxiety
- better
- day
- depression
- doctor
- effect
- feel
- first
- get
- good
- help
- like
- medication
- medicine
- month
- much
- night
- side
- sleep
- started
- still
- take
- taking
- time
- week
- weight
- work
- would
- year

**Top 30 most frequent words in class 2**

- anxiety
- attack
- back
- better
- day
- depression
- doctor
- effect
- feel
- first
- get
- help
- life
- like
- medication
- medicine
- month
- much
- night
- panic
- side
- sleep
- started
- take
- taking
- time
- week
- work
- would
- year

### Uniform Interchangeable Model Fusion

#### Overview

In this study, we not only applied the end-to-end RF [48], SVM [18], and BERT [16] to classify the reviews but also we attempted to optimize the structure of these end-to-end models. We were motivated by the low interpretability of most ML models, which makes it impossible to determine which part of the model plays the most critical role in the model’s performance and which part hinders it. By decomposing these models into standard functional modules and analyzing the performance of each module of those models, we can shed light on the reasons behind their impressive performance for specific NLP tasks. It is then possible to combine the best modules to construct the optimal models. Therefore, we proposed UIMF to fuse different models and displace the structure of the models. It addresses the difference of the underlying algorithmic logic of the neural network and traditional ML end-to-end models and the compatibility between them to fuse functional modules from different types of models. UIMF includes 2 phases: model decomposition inspired by the modularization technique proposed by Kingetsu et al [49], and model recomposition. It decomposes a model into manageable modules based on their functionality. Model recomposition is designed with a compatible model structure that chains the encoder and the classifier sequentially, and the modules are recomposed into fused models.

#### Model Decomposition

In NLP, both traditional ML and neural network models use the encoders to extract and vectorize text features and use the classifiers to perform classification. However, traditional ML models have more modular and independent components than neural network models. Traditional ML models and neural network models have their own strengths: traditional ML models are more robust, interpretable, and computationally efficient, whereas neural network models are better at fitting continuous functions, extracting features, and adapting to different tasks. To the best of our knowledge, no research has proven that the model structure of SVM, RF, or BERT is optimal. Therefore, we attempted to fuse them to combine their advantages from a structural perspective. However, because the structure of the model is contextually linked, randomly splicing and overlaying different model structures may cause internal inconsistencies. Therefore, we applied UIMF to decompose a model into 2 functional modules: the encoder and the classifier. For RF and SVM models, the encoder was a word-embedding algorithm, and the classifiers were the trees and support vectors, respectively. For BERT, the encoder was the multihead attention layers and the feed-forward network layers, and the classifier was the subsequent neural network classification layer. After model decomposition, we obtained 2 encoders—the word-embedding encoder and the transformer bidirectional encoder—and 3 classifiers—RF classifier, SVM classifier, and FCNN classifier—as shown in Figure 1.

For the word-embedding encoder, all the nonrepeating words in the data set were extracted and mapped to a vector space, and the words in the paragraph were replaced with the corresponding vectors. As mentioned earlier, we found that the most frequent words in class 2 were similar to those in class 0 and class 1. However, they lack words with emotional polarity, which may indicate the weak importance of context. To select and weigh the significant words rather than just high-frequency words, we used TF-IDF [11], a statistical measure of word importance in documents. The TF-IDF is combined with word embeddings to create document embeddings, which are vector representations of sentences or documents that preserve the semantic and syntactic properties of words. We multiplied the word’s TF-IDF score by its embedding vector and averaged the result over all the words in the sentence. Thus, we avoided ignoring words with high emotional polarity and weakening their influence on the classification results.

Transformer bidirectional encoders are multihead attention layers and feed-forward network layers extracted from lightBERT. Compared with BERT, lightBERT can meet the requirements of a realistic production environment with edge devices. LightBERT was pretrained on Wikipedia using TensorFlow [50], and we fine-tuned it using the target data set.

As for classifiers, the FCNN layer, RF, and SVM are decomposed as the classifiers. The RF classifier can handle high-dimensional data and nonlinear relationships, perform feature selection and importance ranking, and be robust to noise from few grammar and spelling errors. The SVM classifier can work well with small data sets and be robust to outliers. While the FCNN can learn complex patterns and nonlinear relationships from data and be flexible to different architectures [51]. These classifiers use the decision tree ensemble method, kernel method, and approximation theorem, respectively, for classification, and they are well contrasted with each other.

These encoder and classifier modules are in preparation for the model structure reorganization fusion in the next section to explore whether substituting and recomposing the internal structure of the end-to-end traditional ML and neural network models results in better models.

#### Model Recomposition

**Figure 3:**
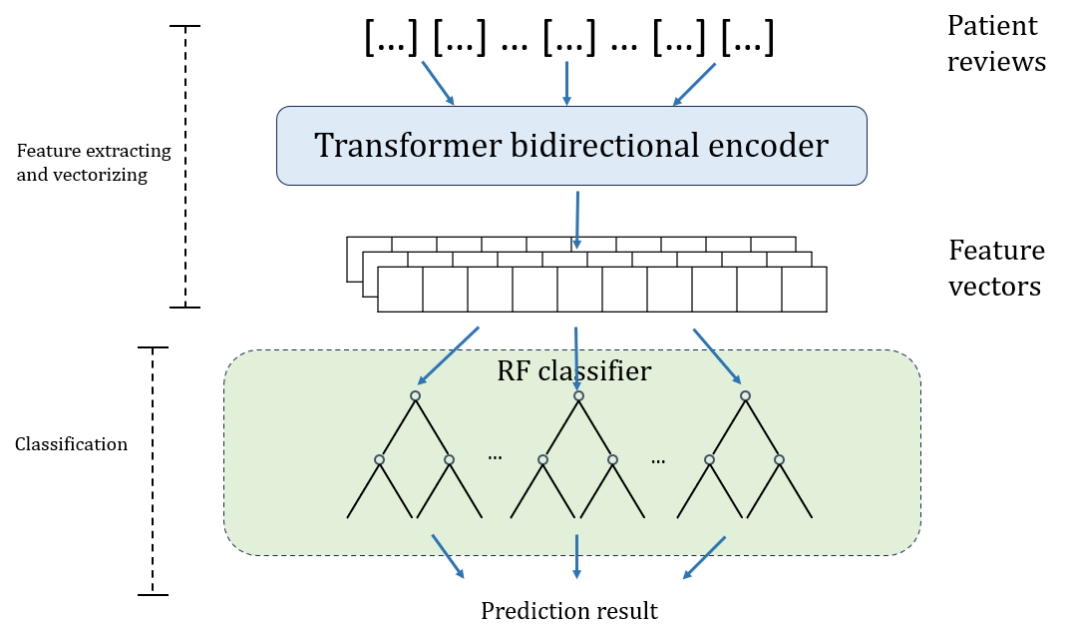
Architecture of transformer bidirectional encoder+random forest (RF) model.

**Figure 4:**
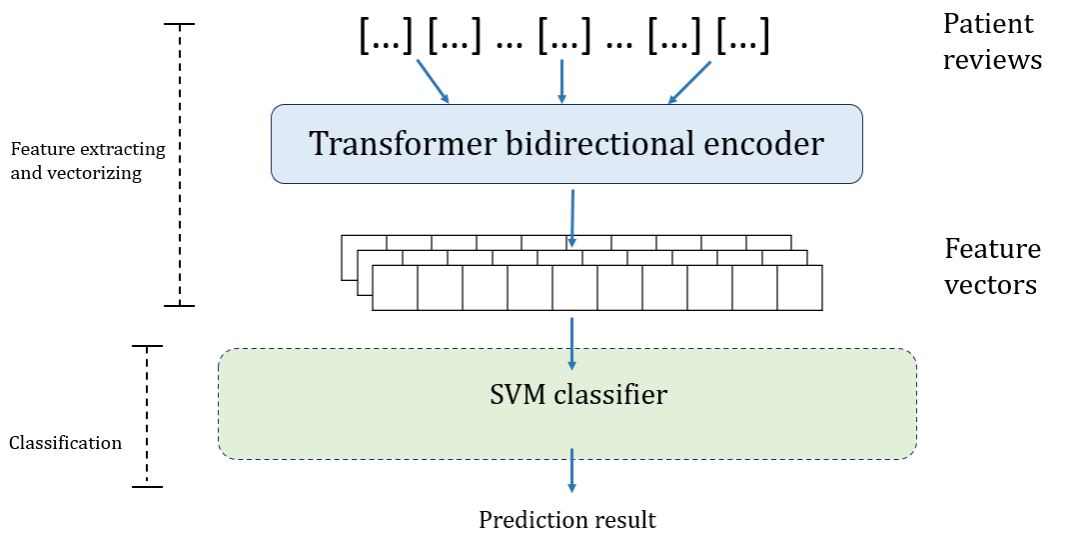
Architecture of transformer bidirectional encoder+support vector machine (SVM) model.

**Figure 5:**
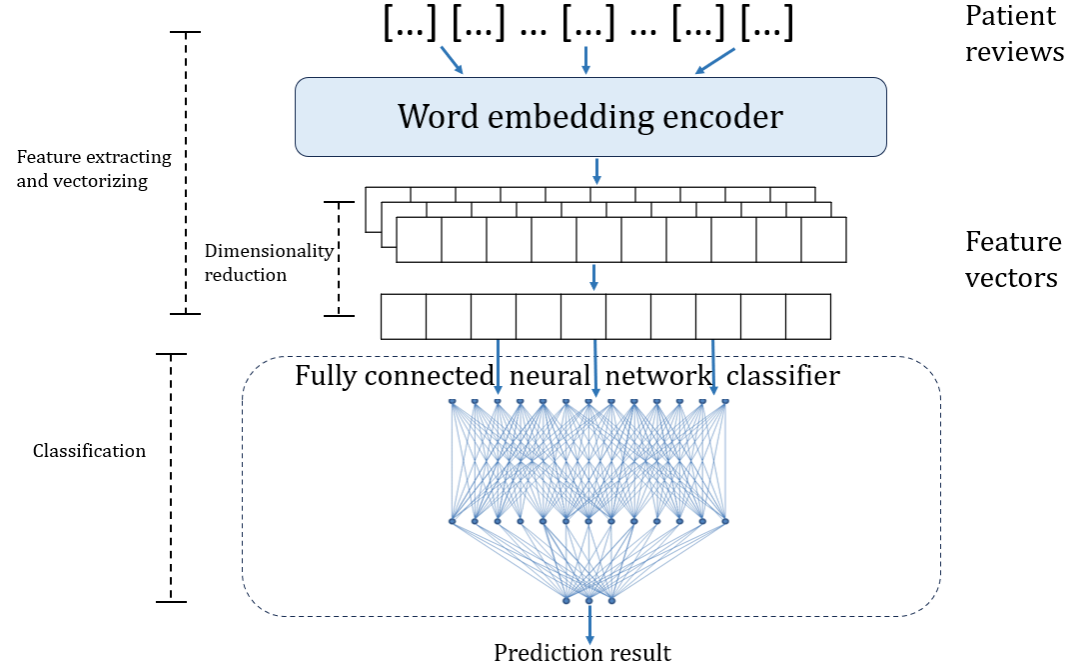
Architecture of the word-embedding encoder+fully connected neural network model. RF: random forest.

In model decomposition, the 2 encoders and 3 classifiers are cross-combined separately to be fused into 6 models. Three of them are existing models from the perspective of model structure: BERT (transformer bidirectional encoder+FCNN), RF (word-embedding encoder+RF), and SVM (word-embedding encoder+SVM). The other 3 models are real fused models including the transformer bidirectional encoder+RF classifier, transformer bidirectional encoder+SVM, and word-embedding encoder+FCNN; and their model structure are shown in Figures 3-5.

As less significant words were not filtered in the word-embedding encoder, which leads to a large number of features that have little impact on the classification result being embedded. Dealing with those high-dimensional feature vectors consumes a large amount of computational resources. To reduce dimensionality, we conducted feature engineering, which scored the importance of feature dimensions based on the TF-IDF and filtered the 6000 most significant dimensions for classification. On the basis of the model structure, the fused models are classified into 2 categories: traditional ML models and neural network models, which consist of hybrid models and pure neural network models.

The traditional ML models include word-embedding encoder+SVM and word-embedding encoder+RF classifier. Imbalanced data will have little effect on the performance of the word-embedding encoder. The SVM classifier attempts to find the optimal hyperplane that maximizes the margins between different classes of feedback from patients. This means that it focuses on patient instances close to the decision boundary (support vector) and ignores those far from the boundary. Consequently, it is less affected by the majority class. The RF classifier is also less affected by noise and outliers, which may be present in the majority classes. It builds multiple decision trees based on different classes of feedback from patients and their features to reduce the correlation and variability between trees and to increase the diversity and robustness of the set of trees. Overall, these 2 models are robust to imbalanced data sets.

The neural network models include the word-embedding encoder+FCNN classifier, transformer bidirectional encoder+SVM classifier, transformer bidirectional encoder+RF classifier, and transformer bidirectional encoder+FCNN classifier. The difference between BERT and our transformer bidirectional encoder+FCNN model is that the loss function of BERT model is cross-entropy, whereas the loss function of our transformer bidirectional encoder+FCNN is the focal flooding (FF) loss. In general, the loss function that the neural network models share is cross-entropy loss. Thus, for gradient-based optimization methods, the errors from the majority class (class 2) will predominate over the errors from the minority classes (class 0 and 1) and have a more significant impact on the parameter adjustment. To add insult to injury, because too many parameters and layers are used to boost the complexity and adaptability of these models, they can easily overfit to the majority class and underfit to the minority class. This situation can lead to poor generalization and low recall for the minority classes, which is more important in the target data set. We address this problem by modifying the loss function. The flooding loss [52] and focal loss [53] that are with high profile from computer vision are borrowed for this task. A new loss function, called FF loss, is defined as follows:



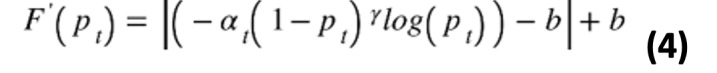



where *b* is a hyperparameter to control the minimum of the loss function against overfitting, is used to change the weight for negative examples (majority samples) to release the data imbalance problem, and γ is used to reduce the weight of easily classified samples to improve the performance of the model on data that are difficult to classify.

Hence, the neural network models with FF loss adjust the weights to focus more on the particular class of patients’ reviews and reviews that are difficult to classify to prevent overfitting. We applied FF loss to all our fused neural network models. Finally, the Adamw [54] optimizer was used to optimize the parameters of the models. In addition, all these models were trained using an 11th Gen Intel Core i9-11900KF@3.50GHz CPU and an NVIDIA GeForce RTX 3080 GPU.

## Results

Extensive experiments were conducted for two main purposes: (1) fine-tuning all the fused models and (2) performance comparison between the fused models.

### Optimization of the Transformer Bidirectional Encoder+FCNN Model

#### Loss Function Adjustment

**Figure 6:**
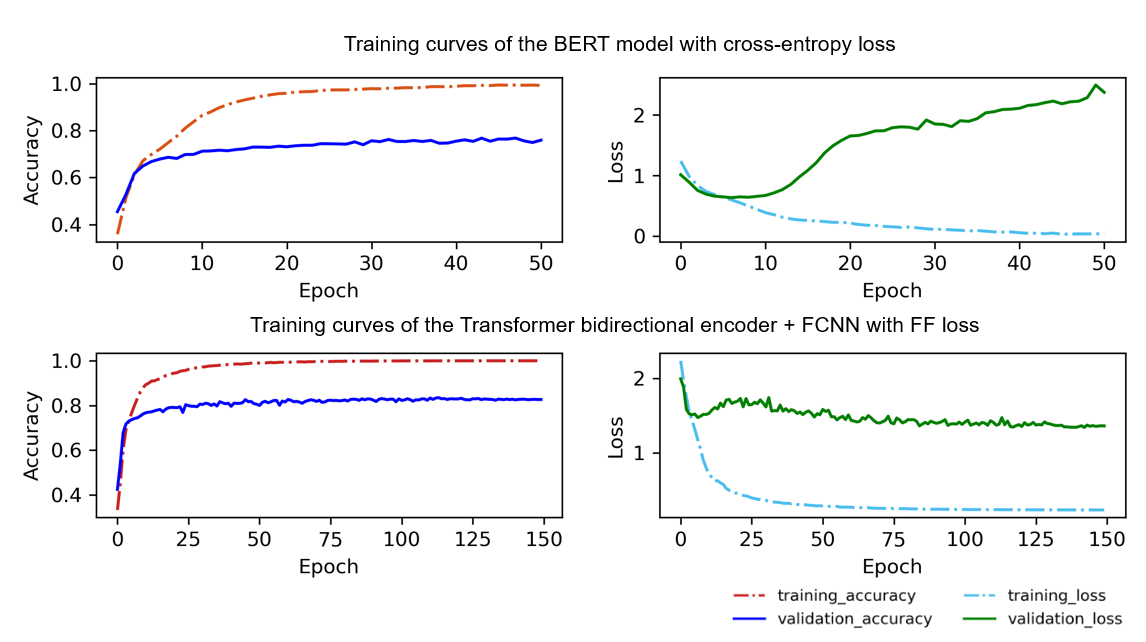
Training curves of the Bidirectional Encoder Representations From Transformers (BERT) model with cross-entropy loss and the transformer bidirectional encoder+fully connected neural network (FCNN) with focal flooding (FF) loss.

As a benchmark, BERT was fine-tuned. The first 2 subfigures in Figure 6 show the training curve of the model with the cross-entropy. An overfitting problem appears even with the early stopping [55] regularization method. It is obvious that after decreasing at the start of the training, the validation loss continues to increase at the later phase of the training, although the other 3 lines continue to behave normally. This could be because the model training is in the early stage of overfitting, and it learns the specific patterns and noise in the training data too well but fails to generalize to new and unseen data. Moreover, the bias is temporarily insufficient to have a significant effect on the other curves for the time being. Hence, the remainder of the curve remains stable. However, as the number of training epoch increases, the model will inevitably learn a large amount of noise, which leads to significant increase in the validation loss and significant decrease in the validation accuracy.

Therefore, we trained the transformer bidirectional encoder+FCNN model (same structure as a BERT model [16]) but with FF loss function. The hyperparameters are set as follows: α=1.0, γ=1.0, b=0.125, and 2 hidden layers of the FCNN are used. The third and fourth subfigures of Figure 6 show that the validation loss rises briefly near the 25th epoch and then declines steadily until it stabilizes, whereas the validation accuracy increases steadily. Moreover, the accuracy of the transformer bidirectional encoder+FCNN model improved by approximately 3% to 6% compared with the BERT model. This was because limits the minimum value of the training loss, which can prevent the model from overlearning to the noise in the data set. It is significant proof that the FF loss function can effectively stop overfitting from occurring.

#### Hyperparameter Adjustment in Loss Function

To optimize the performance, hyperparameter adjustment experiments were conducted on the model with FF loss. The optimization focusing on adjusting *a, γ, b* is just needed to be given a suitable value because is essentially a regularization method, which is mainly for against overfitting. If the overfitting does not exist, regularization may not significantly improve model performance because it indicates that the model already has a good balance between bias and variance.

**Figure 7:**
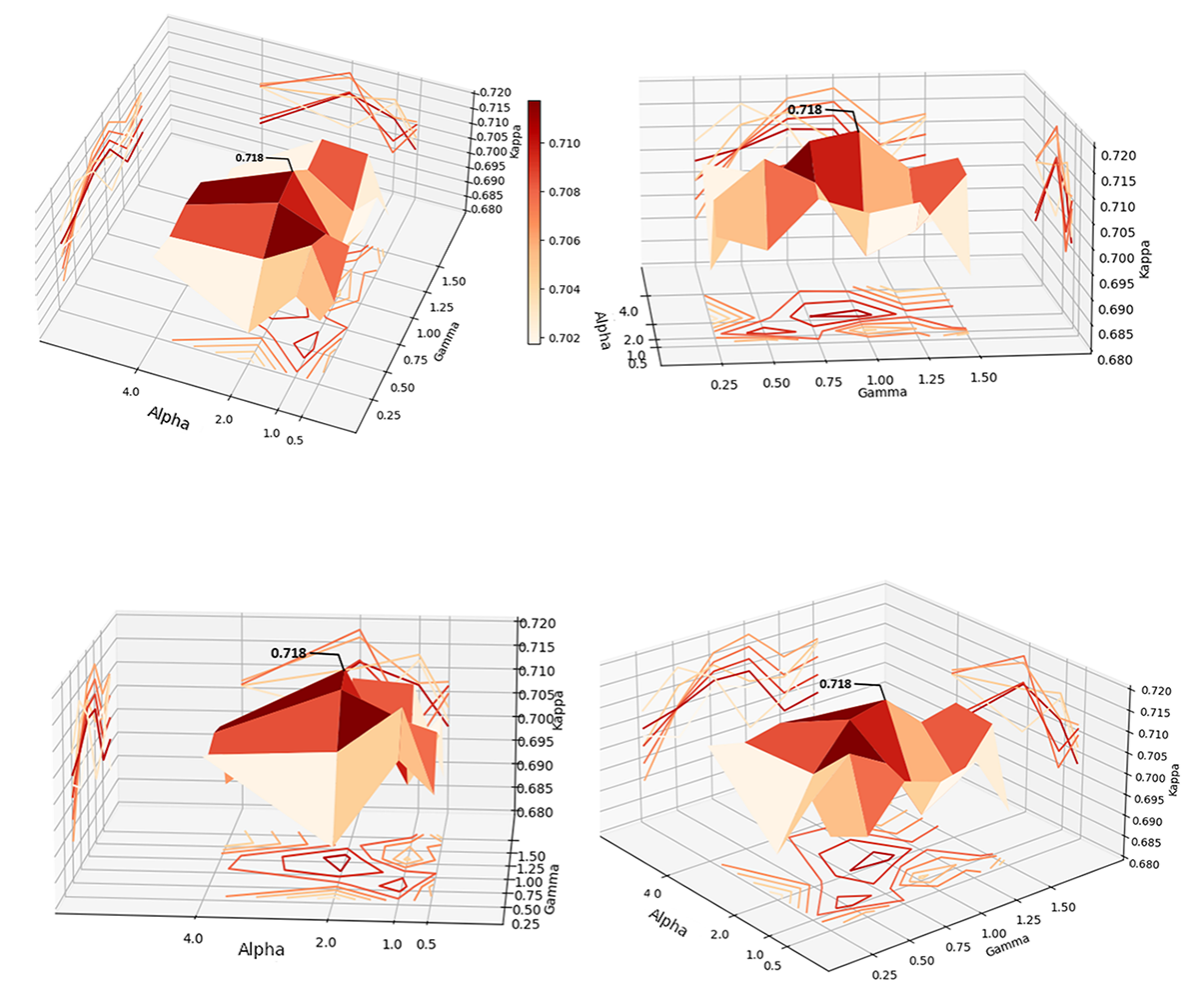
The κ coefficient of the transformer bidirectional encoder+fully connected neural network model with 2 hidden layers.

In these experiments, α of class 1 was adjusted while keeping α of class 0 and class 2 unchanged because the prediction accuracy for this class was much lower than in previous experiments. α of class 1 was set to be 0.5, 1.0, 2.0, and 4.0 and α of class 0 and class 2 were 1.0. In addition, γ was set to 0.25, 0.50, 0.75, 1.00, 1.25, and 1.50, which controls the weight of the data that are easy to classify. We formed 24 sets of α and γ pairs by permutation and experimented with each pair to generate Figure 7.

Figure 7 shows the trend in the κ coefficients of the model with respect to α and γ. The surface is flatter when α is larger than 2.0. The chart has a valley when α=1. As γ increased to 1.0, the surface rises. There is a ridge at γ=1. The peak of the κ coefficient appears when α=2 and γ=1.00−1.25. As we mentioned in FF loss function of model recomposition, is used to change the weight for negative examples (majority samples) to release the data imbalance problem, and is used to reduce the weight of easily classified samples. Hence, optimal model performance can be achieved by setting the weight of class 1 samples to 2 and the weight of difficultly classified samples to a range of 1.0 to 1.25. The number of hidden layers is also tuned; however, it does not significantly affect the κ coefficient.

### Optimization of the Transformer Bidirectional Encoder+RF Model

**Figure 8:**
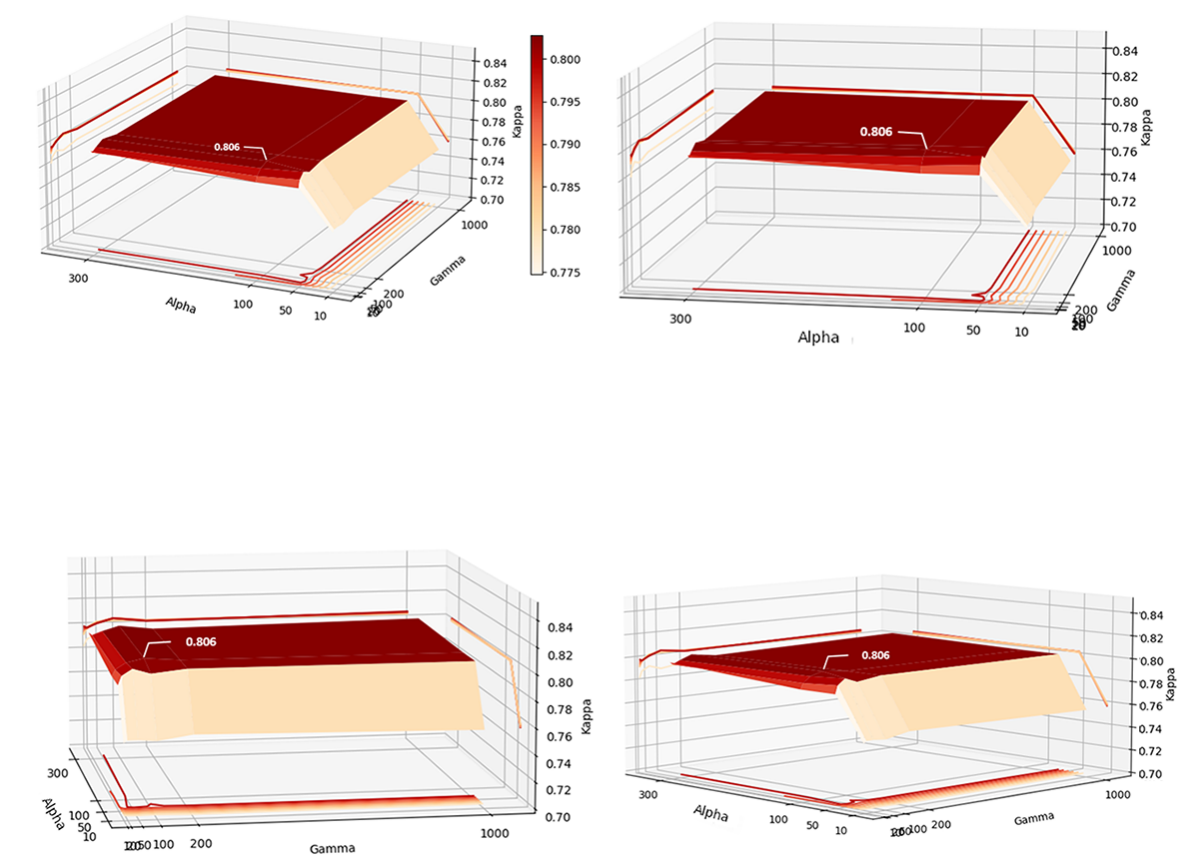
The trend of the κ coefficient of the transformer bidirectional encoder+random forest.

Table 3 shows the average κ coefficient of the models with different maximum depths and different numbers of subtrees. The κ coefficient varied in the range from 0.754 to 0.809. The trend of the κ coefficient with respect to the number of subtrees (γ) and maximum depth (α) is shown in Figure 8. It can be observed that the performance increases when increasing both gamma and alpha before they reach 100. After 100, increasing both alpha and gamma significantly increased the computational cost and reduced the generalizability of the model. The optimal model is with the maximum depth of 100 and 100 subtrees.

**Table 3:** The κ coefficient of the transformer bidirectional encoder+random forest with different number of subtrees and maximum depth.

Maximum depth	Number of trees					
	10	20	50	100	200	1000
10	0.754	0.756	0.756	0.755	0.759	0.756
50	0.791	0.798	0.803	0.799	0.802	0.802
100	0.791	0.796	0.801	*0.809* ^a^	0.802	0.802
300	0.799	0.797	0.800	0.804	0.801	0.804

^a^The highest κ coefficient is italicized.

### Optimization of the Transformer Bidirectional Encoder+SVM Model

We compared the performance of the models with linear and Gaussian kernels on the validation set. The linear kernel showed better results than the Gaussian kernel. The linear kernel showed better results than the Gaussian kernel in terms of *F*_1_-score, accuracy, precision, recall, and κ coefficient. The linear kernel achieved an *F*_1_-score of 0.833, an accuracy of 0.837, a precision of 0.833, a recall of 0.837, and a κ coefficient of 0.731. The Gaussian kernel, on the other hand, achieved an *F*_1_-score of 0.826, an accuracy of 0.829, a precision of 0.825, a recall of 0.829, and a κ coefficient of 0.720. Besides, the model with a linear kernel achieves faster training, faster prediction, and lower cost, but its prediction accuracy for linearly indistinguishable data is much lower than that with a Gaussian kernel. The results indicate that the patients’ reviews processed by the transformer bidirectional encoder are highly linearly separable.

### Optimization of the Word-Embedding Encoder+FCNN Model

The FCNN classifier was extracted from the fine-tuned transformer bidirectional encoder+FCNN model and then recomposed with the word-embedding encoder. Because the output of patients’ reviews processed by the word-embedding encoder is up to 6000 dimensions, the number of input features of FCNN classifier increases significantly. Therefore, we increased the number of neurons in each hidden layer of the FCNN classifier. The best model achieved a κ coefficient of 0.573 when α=2 and γ=1.5. This model performs much worse than the other fused models.

### Optimization of the Word-Embedding Encoder+RF Model

**Figure 9:**
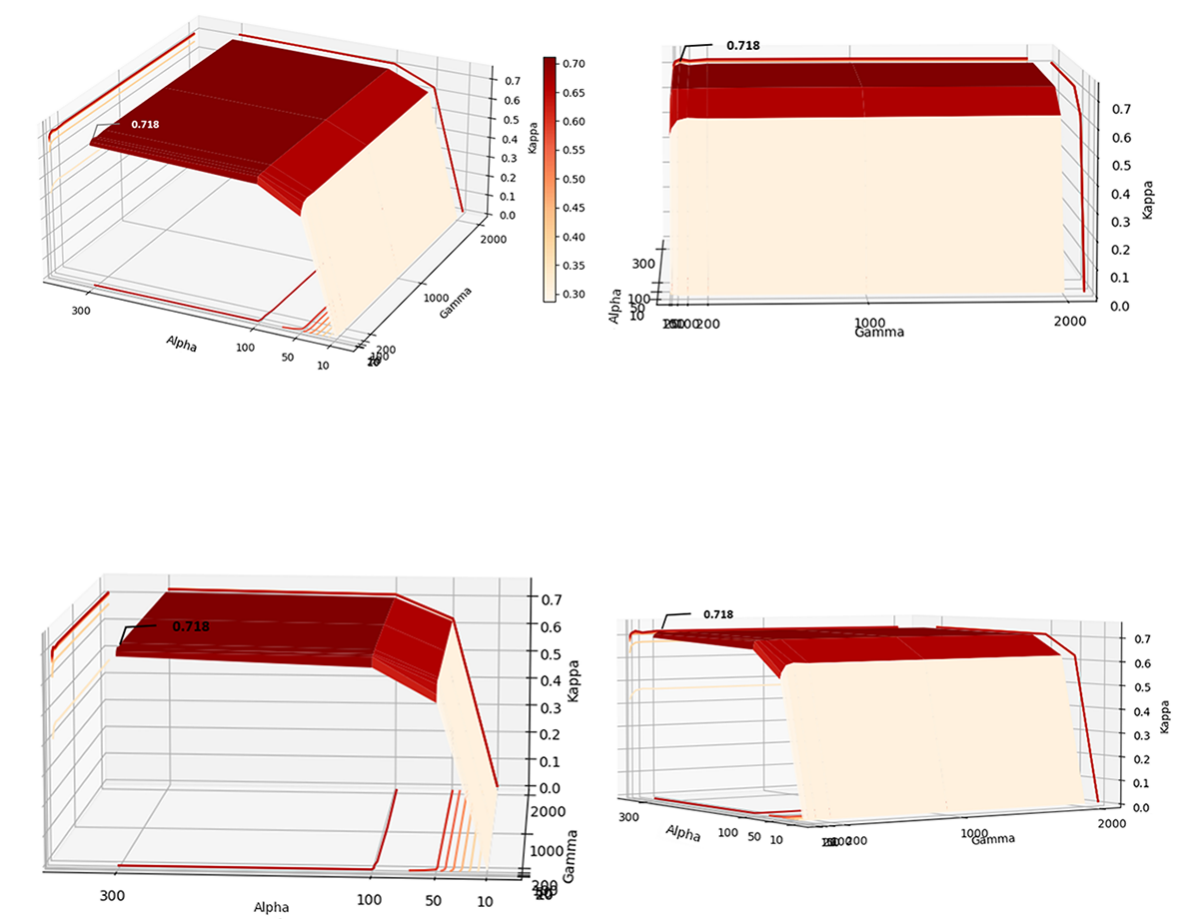
The κ coefficient of the word-embedding encoder+random forest model.

Because the input of this model was up to 6000 dimensions, the maximum number of subtrees in the experiments was increased to 2000. The trend of the κ coefficient with respect to number of subtrees (gamma) and maximum depth (alpha) is shown in Figure 9, as increasing the number of subtrees after reaching 50 and the maximum depth after reaching 300 does not significantly improve κ coefficient, but significantly increases the computational cost and reduces the generalizability of the model, the optimal word-embedding encoder+RF model is the model with a maximum depth of 300 and 50 subtrees, reaching a κ coefficient of 0.718.

### Optimization of the Word-Embedding Encoder+SVM Model

We compared the performance of the model using the linear and Gaussian kernels on the validation set. The linear kernel achieved an *F*_1_-score of 0.701, an accuracy of 0.708, a precision of 0.700, a recall of 0.708, and a κ coefficient of 0.514. The Gaussian kernel, on the other hand, achieved an *F*_1_-score of 0.824, an accuracy of 0.824, a precision of 0.825, a recall of 0.824, and a κ coefficient of 0.715. It can be observed that all metrics of the Gaussian kernel are higher than those of the linear kernel. The results may also indicate that the patients’ reviews processed by the word-embedding encoder are highly linearly indivisible.

### Model Comparisons

**Figure 10:**
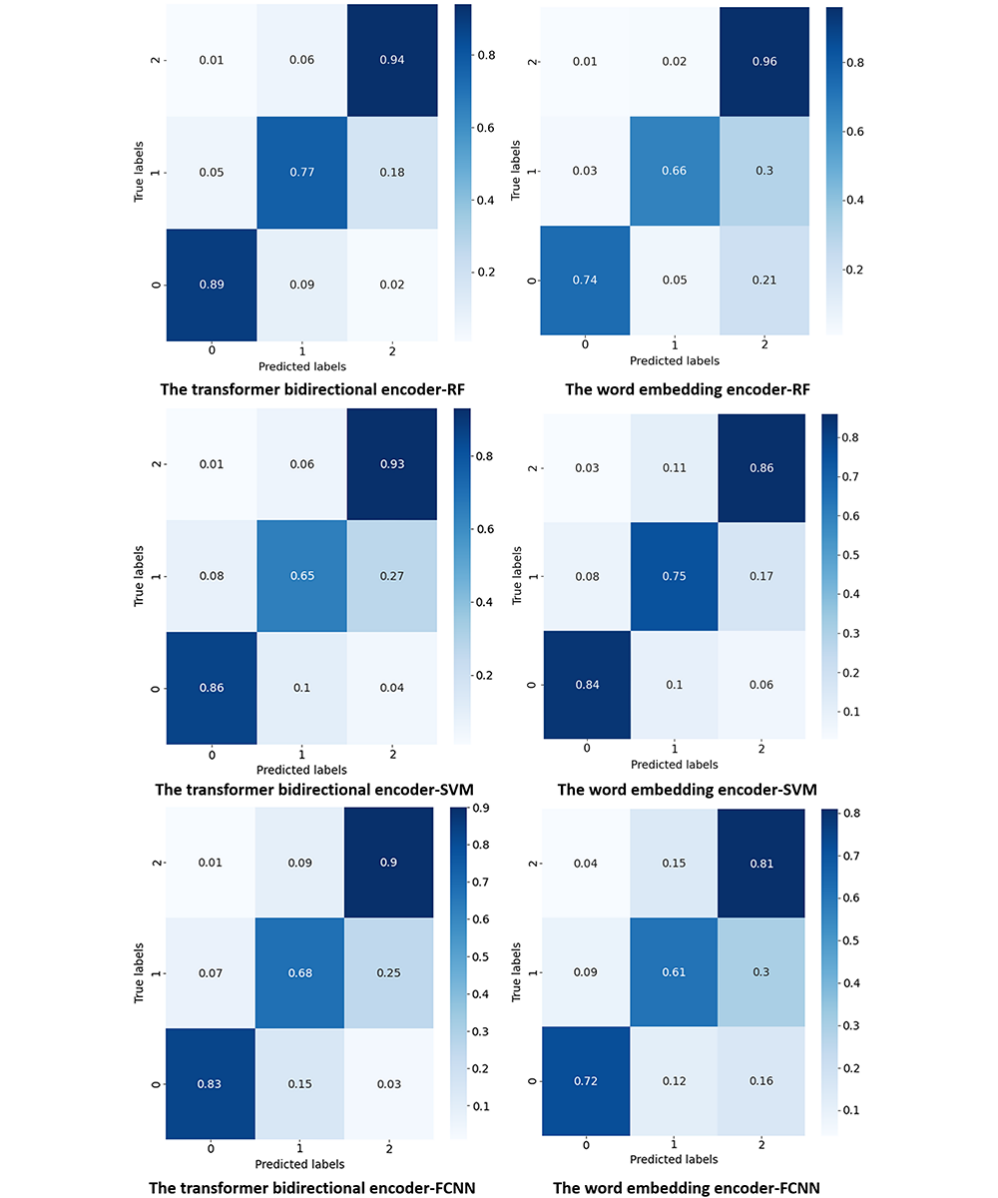
The confusion matrix of recomposed models. FCNN: fully connected neural network; RF: random forest; SVM: support vector machine.

A comprehensive comparison is made among all the optimized and fused models in terms of the average graded *F*_1_-scores, accuracy, κ coefficient, and training time in Table 4. The models were evaluated using 10-fold cross-validation. In addition, results were obtained based on the average value of 10 times testing. The graded *F*_1_-score is a metric derived from a knowledge-aware assessment of the severity of suicide risk for early intervention. The weights of the 3 classes are 2, 1 and 1, because class 0 is more important than class 2. Figure 10 shows the confusion matrices of all the optimized fused models. The accuracy for predicting each class varies in different models. In general, the transformer bidirectional encoder+RF model achieved the best overall performance, reaching a graded *F*_1_-score of 0.872 and a κ coefficient of 0.806 at the expense of more training time. According to Figure 10, its accuracy for the 3 classes is balanced where class 1 is with the lowest accuracy of 0.77 and class 0 and class 2 are with high accuracy of 0.89 and 0.94, respectively. The word-embedding encoder+RF is the fastest model, which only required 97.635 seconds for training, and its performance is reasonably acceptable. It achieves a graded *F*_1_-score of 0.801 and the κ coefficient of 0.695. The corresponding confusion matrix in Figure 10 shows that the model has a particularly good accuracy for class 2 but poor accuracy for class 1.

**Table 4:** Comparison of the optimized models.

Fused models	Graded *F*_1_-score	Accuracy	κ	Train times
Transformer bidirectional encoder+RF^a^	*0.872* ^b^	*0.873*	*0.806*	45,357.947
Transformer bidirectional encoder+SVM^c^	0.861	0.863	0.775	45,311.353
Transformer bidirectional encoder+FCNN^d^	0.857	0.858	0.769	45,297.559
Word-embedding encoder+RF	0.801	0.812	0.695	*97.635*
Word-embedding encoder+SVM	0.797	0.806	0.687	5233.945
Word-embedding encoder+FCNN	0.734	0.735	0.577	24,196.99
BERT^e^ (benchmark) [16]	0.846	0.846	0.746	45,382.829
MentalBERT	0.867	0.867	0.785	110,669.089

^a^RF: random forest.

^b^Optimum value.

^c^SVM: support vector machine.

^d^FCNN: fully connected neural network.

^e^BERT: Bidirectional Encoder Representations From Transformers.

## Discussion

### Principal Findings

In Table 4, regarding graded *F*_1_-scores, accuracy, and κ coefficient, the 3 fused models using the transformer bidirectional encoder have better overall performance than those using the word-embedding encoder. The models with the transformer bidirectional encoder have more stable and balanced performance. This is because the transformer bidirectional encoder can map patients’ reviews in a low-dimensional but more linearly separable vector space. Among the classifiers, the RF classifier is the best classifier. The reason behind this is it averages the results of multiple decision trees, each trained on a different subset of the data, to produce a more robust and accurate prediction. RF exhibits better performance, especially with the transformer bidirectional encoder.

The transformer bidirectional encoder+RF model achieves the best average performance, which far exceeds the state-of-the-art BERT model. The corresponding confusion matrix shows that the model performed the best accuracy in class 2, reaching 0.94. The lowest accuracy of 0.77 is for class 1. The reason is that the amount of data in class 2 is much larger than that in class 1, and the language style of the reviews in class 1 is more ambiguous without distinctive features. In addition, the transformer bidirectional encoder+RF model showed better performance on every metric and only 2 out of 5 of the training time compared with the current state-of-the-art MentalBERT model in the mental health domain. The variation in accuracy for all classes was relatively small compared with the other models. It is worth mentioning that the κ coefficient reaching 0.81 indicates the prediction results of the model are almost in perfect agreement with the random choice of patients’ subjective satisfaction [34]. It means our model can precisely evaluate the satisfaction of the treatment. However, this method especially consumes graphics processing units, storage, and time resources. It may require the psychotherapy and mental drug treatment providers to have sufficient computing resources to train their patient reviews.

However, for the word-embedding+RF model, the training time decreases significantly at the expense of decreasing the graded *F*_1_-score by 0.071 and accuracy by 0.061. If considering a practical scenario with limited resources available for training, such as small clinics or small hospitals, the word-embedding encoder+RF is also a good choice for a fast implementation.

### Implications for Clinical Use

This pilot study is an in-depth exploration of AI methods for assisting psychologists in developing or optimizing mental medication regimens by analyzing the patients’ satisfaction with mental drug treatment and reducing the impact of mental disorders on global development and alleviating the problem of strained mental health care resources in the post–COVID-19 pandemic era. The transformer bidirectional encoder+RF model outperformed the state-of-the-art BERT model and MentalBERT model in most aspects, and in terms of training time, it is comparable to BERT and significantly better than MentalBERT. Hence, we showed that the transformer bidirectional encoder+RF model fused via UIMF is one of the best models for predicting patients’ satisfaction. In addition, for environments with constrained computational resources, the word-embedding encoder+RF could be used for a much faster training time. Both have the potential to assist psychologists in analyzing patients’ satisfaction with drug treatment, while increasing their efficiency in the clinical setting. Moreover, it also has the potential to serve as a significant tool for the training and advancement of trainee psychologists.

### Limitations

There are still some limitations to be addressed in future studies. For instance, the spelling and grammar corrections in this study were slightly deficient in terms of performance, which might have inevitably introduced noises. Moreover, the best model relied on a large pretrained language model that might contain biases or errors, and it may negatively affect the quality of the prediction. Hence, we suggest that future work can focus on exploring the potential of the GPT-4 model for spelling and grammar correction during data preprocessing. In addition, future attempts could be made to simulate patients using the GPT-4 model to generate more comprehensive textual data of the mental disorders health care domain based on existing limited data to train language models and improve the robustness of pretrained language models. Moreover, future work can attempt parallel fusion of multiple feature extractors, including BERT, BioMegatron, and GPT models, whose outputs are separate modalities and allow all modalities to be fused at the input level of the predictor, which is then learned by the predictive model.

### Comparison With Prior Work

After a thorough search of previous studies, there are not many relevant studies. A study [56] investigated the performance of the n-gram, RNN, and BERT on their data set related to psychotherapy. Their results showed that the BERT model performed better than the other models. They did not attempt to optimize the performance of the present classifier, and their task only focused on DL models and simply classified the sentiment into 3 classes. However, in this study, we classified data with a more reasonable protocol, proposed a new loss function, and fine-tuned both ML and DL models. In another study [57], a new framework for evaluating a sentiment analysis model was developed. They trained CNN, LSTM, and gated recurrent units on a data set of movie reviews. The gated recurrent unit shows more explainable results related to psychological states. In contrast to this study, our models were trained on the data set of reviews from patients with mental illness, so the prediction results were more interpretable and plausible. Besides, a dictionary of medical opinions was built in the study by Asghar et al [24]. They were based on the corpus of health reviews and a medical polarity lexicon. The sentiment score was then generated by computing the word polarity score of the text data. This is a traditional and less-efficient approach, whereas our research explored advanced AI-based methods. Overall, they mechanically used various existing models without attempting to reorganize the model structure and optimize the model performance, and they did not relate the patient’s emotional state to the treatment outcome. Our proposed approaches are better because we not only explore existing models but also design the UIMF approach and the FF loss function to construct fused models for optimization. Moreover, through our investigation, we provide practical suggestions on where to apply those models in clinical scenarios using different computing resources.

### Conclusions

This study aimed to provide effective and accurate classification models to evaluate mental drug treatment satisfaction using reviews of patients with a mental disorders. The data set consisting of reviews of 20 disorders related to mental health was extracted from the Kaggle data sets and then preprocessed. We proposed the UIMF method, which decomposes state-of-the-art BERT models and traditional ML models into corresponding encoders and classifiers, followed by recomposing them to form 6 fused models. On the basis of our experiments, an optimal model with the highest accuracy and a suboptimal model with fast training and fair accuracy are obtained and these can be applied in different scenarios.

Our work bridges this gap in this field, and its results can be considered as a valuable reference for psychologists. In addition, it opens up new possibilities for alleviating the conflict between the large increase in patients with mental illness and insufficient medical resources. By combining RF and transformer models, the contextual information of patients’ reviews can be used to achieve better accuracy. Meanwhile, the model that combines RF and the word-embedding encoder meets the requirements of the production environment, in which the speed and efficiency of devices are limited, and the time resource is insufficient.

We believe that this research will contribute to the advancement of AI as a core method for improving mental illness treatment. DL and ML methods can analyze patients’ feedback and measure their satisfaction with mental drug treatment, which can significantly improve the productivity of psychologists as well as treatment outcomes.

## Abbreviations

AI: artificial intelligence
BERT: Bidirectional Encoder Representations From Transformers
BOW: bag-of-words
CNN: convolutional neural network
DL: deep learning
FCNN: fully connected neural network
FF: focal flooding
LSTM: long short-term memory
ML: machine learning
NLP: natural language processing
RF: random forest
RNN: recurrent neural network
SVM: support vector machine
TF-IDF: term frequency–inverse document frequency
UIMF: Unified Interchangeable Model Fusion
